# Adipose–Cardiac Crosstalk in Obesity-Related Atrial Fibrillation: The Emerging Role of Extracellular Vesicles

**DOI:** 10.1016/j.jacbts.2026.101598

**Published:** 2026-06-22

**Authors:** Naveed Akbar, Svetlana Reilly

**Affiliations:** Division of Cardiovascular Medicine, Radcliffe Department of Medicine, University of Oxford, Oxford, United Kingdom

**Keywords:** adipose tissue, atrial fibrillation, cardiac remodeling, extracellular vesicles, obesity

**Figure:**
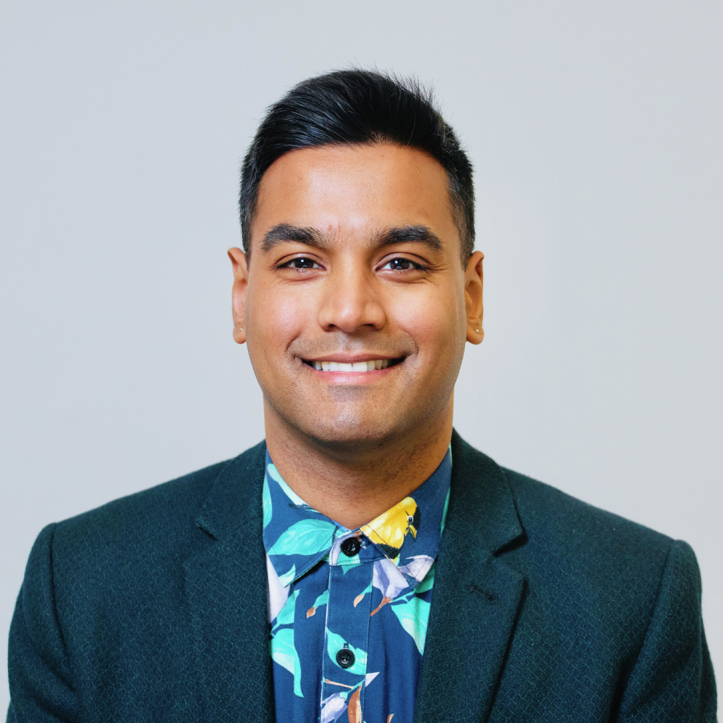

**Figure:**
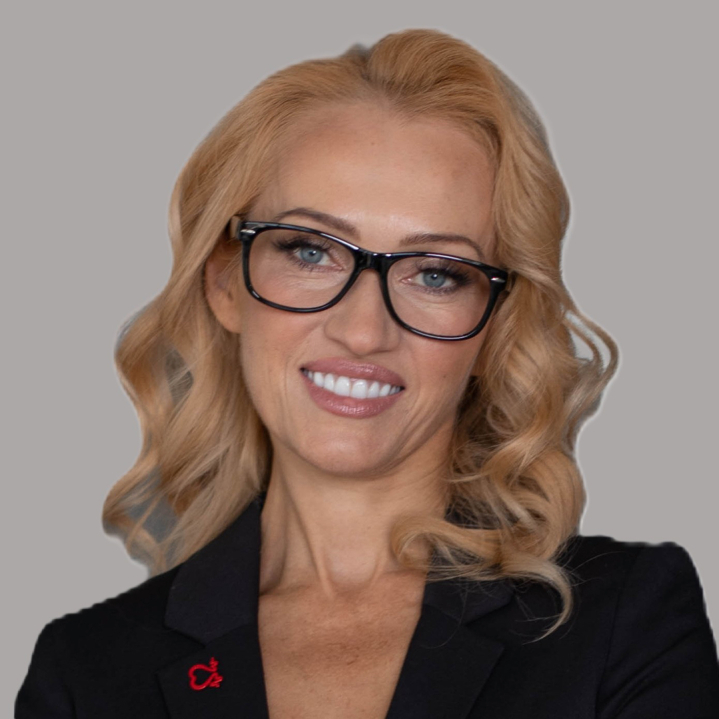

Overweight and obesity, now affecting approximately 2.5 billion adults worldwide, have been accompanied by a parallel rise in cardiovascular morbidity.^1^ Among its most clinically challenging complications are cardiac arrhythmias, specifically atrial fibrillation (AF) and sudden cardiac death. A high body mass index is associated with an increased risk of AF and with atrial structural and electrical remodeling that promotes arrhythmogenesis; however, the precise molecular interactions between adipose tissue and the myocardium remain incompletely understood.

In this issue of *JACC: Basic to Translational Science*, Limpitikul et al^2^ explored this interaction. By integrating human electrophysiology, atrial induced pluripotent stem cell (iPSC)–derived cardiomyocytes, and a sophisticated in vivo genetic extracellular vesicle (EV) tracking model, the authors propose that visceral fat does not just act as a passive metabolic burden, but also actively communicates with multiple myocardial cell types (cardiomyocytes, fibroblasts, and macrophages) via EVs. This interaction leads to the electrical and structural atrial remodeling.

The established model of obesity-associated AF emphasizes systemic mechanisms such as hemodynamic load, sleep apnea–related autonomic changes, and chronic inflammation, alongside local adipose tissue-mediated effects within the atrium. However, these factors alone do not fully explain the more advanced atrial remodeling and reduced effectiveness of rhythm control strategies, including catheter ablation, observed in obese patients.

Although adipose-cardiac crosstalk is well recognized, the precise mechanisms by which adipose-derived signals are delivered to and act on atrial cells, particularly the role of EVs and their in vivo relevance and contribution to atrial arrhythmogenesis, are incompletely defined.

Limpitikul et al^2^ further refine this concept by emphasizing a more direct vesicle-mediated mechanism of interorgan communication. The study focuses on how visceral adipose tissue–derived extracellular vesicles (VAT-EVs) can reach cardiac cells and modulate their functional and transcriptional phenotype.

A major strength of this paper is that it does not focus solely on the effects of EVs on cardiomyocytes, and also explores their impact on other key cell types involved in the arrhythmic process, including fibroblasts and macrophages.

The study reports prolongation of action potential duration (APD) in human atrial myocytes from obese patients, a finding that was recapitulated in iPSC-derived atrial cardiomyocytes following exposure to VAT-EVs from obese donors. While this differs from experimental models of AF, which often demonstrate APD shortening, it highlights the complexity of electrophysiological remodeling in obesity. These findings suggest that obesity-related AF may represent a distinct substrate, with potential implications for therapeutic strategies compared with AF associated with other conditions such as hypertension.

Structural remodeling, hallmarked by atrial fibrosis, is a major component of AF, and this study also demonstrates that VAT-EVs trigger a profibrotic response, inducing the differentiation of cardiac fibroblasts into myofibroblasts and significantly increasing collagen production. Thus, VAT-EVs may impact atrial structural substrate for AF in these patients.

Inflammation is an important pathogenic mechanism in AF. Interestingly, the authors show that VAT-EVs are capable of shifting cardiac macrophages toward a proinflammatory state (CD206-negative), both in cell culture and in obese mouse models. This local inflammation may further exacerbate the arrhythmogenesis in the obesity setting.

Another important observation of this work is the identification of a specific, druggable ion channel target TRPC3. Through RNA sequencing and transcriptome-wide association studies, the authors found that TRPC3, a nonselective cation channel, is significantly upregulated by VAT-EV exposure. Crucially and translationally relevant, they demonstrated that a small-molecule inhibitor of TRPC3 (Pyr3) could rescue the APD prolongation, providing a new basis for future new pharmacological interventions.

The use of the Exomap1 mouse model provides in vivo evidence that adipose-derived EVs can reach the heart and cardiac cells to mediate effects in cardiomyocytes in obese states. This supports that the mechanisms observed in vitro are replicated in vivo.

Despite the study’s comprehensive exploration, several areas remain to be further clarified and investigated.

First, while the study establishes that VAT-EVs reach the heart and induce pathological changes, it does not definitively identify the specific cargo within EVs, and which molecules (microRNA, metabolites, and/or ions), or their combination, are responsible for the upregulation of TRPC3 or the activation of fibroblasts and macrophages. The exact signaling molecules on or within EVs remain unknown but would be of particular interest to future therapeutic developments.

Next, although iPSC-derived cardiomyocytes are a powerful human-derived tool, they are developmentally immature compared with adult myocytes. The authors themselves acknowledge that the ionic current kinetics of these cells may not perfectly mirror the complex electrophysiology of an adult human heart. This maturity gap means that while the direction of the APD change is clear, the magnitude and exact phase of the electrical disturbance might differ in a clinical setting. A validation of these findings in human adult atrial cardiomyocytes would be necessary for further validation of this phenotype.

Furthermore, the acute nature of the experimental exposure (24-96 hours) may not fully capture the cumulative impact of a long-standing obesity usually seen in the clinical setting. Moreover, while the study demonstrates that exposure causes changes in cardiomyocytes, it does not address whether these changes are reversible through weight loss, or whether permanent epigenetic shifts in adipose tissue^3^ are conveyed via EVs as an arrhythmogenic substrate.

Last, the study focuses on the right atrial appendage for its human tissue samples. While this is the most accessible tissue during surgery, the primary triggers for AF often reside in the pulmonary veins or left atrium. Whether the adipose tissue–heart communication axis operates with the same intensity or through the same ion channels and mechanisms in these other sites remains to be seen, in addition to whether the dynamic of these changes might be more pronounced in left atria, as it is the most remodeled in AF.

Overall, while targeting EV pathways is conceptually appealing, translating this into safe and effective therapies remains a significant challenge.

The findings of Limpitikul et al^2^ open several exciting avenues for future research and clinical translation. For example, future studies should focus on high-throughput multiomics of the VAT-EV cargo in order to identify specific RNAs, proteins, metabolites, or other cargo unique to obesity and obesity-AF populations. Macrophages are primary effector cells in the capture of adipose tissue–derived EVs in metabolic disease^4^ and can prime the myocardium against oxidative damage following reperfusion injury through the transfer of mitochondria.^5^ These molecules may serve as circulating biomarkers to identify which obese patients are at the highest risk for sudden death or AF before an event occurs.

This study also raises a clinically important question—whether these EV-induced changes are reversible. Investigating EV profiles in patients, for example, after bariatric surgery or after significant weight loss (eg, via glucagon-like peptide-1 agonists) could help to understand if the communication between adipose tissue and the heart can be mitigated or silenced and if the arrhythmogenic substrate can reverse once the obese EV burden is reduced.

Given that TRPC3 inhibition rescued the phenotype, clinical trials or ex vivo human heart studies could explore TRPC3 as a novel antiarrhythmic target specifically for the obese population. This may help to develop a more tailored therapeutic approach in which the treatment for AF is dictated by the patient’s underlying metabolic phenotype observed in patients with obesity.

Utilizing advanced organoid technologies incorporating adipose tissue and multiple cardiac cell types would further help to better replicate the chronic interorgan communication described in this paper and would potentially provide new insights on the paracrine changes within multiple cell types.

In summary, Limpitikul et al^2^ present an elegant study that advances understanding of adipose–cardiac communication in AF. The identification of EVs as mediators of this interaction represents a biologically meaningful mechanistic refinement to the understanding of myocardial remodeling in complex pathology such as AF. While the findings are broadly consistent with current concepts of AF as a disease of structural, inflammatory, and electrophysiological remodeling, some observations require careful interpretation, especially considering multiple confounders in patients with obesity and AF. Moreover, future work is needed to decode the specific molecules/messengers within these vesicles. Overall, the study provides a valuable contribution to the field of obesity-associated arrhythmogenesis by highlighting the complexity of the adipose tissue–heart interaction and points toward new directions for mechanistic research and potential therapeutic development into metabolic-focused therapies.

## Funding Support and Author Disclosures

The authors have reported that they have no relationships relevant to the contents of this paper to disclose.

## References

[1] NCD Risk Factor Collaboration (NCD-RisC) (2024). Worldwide trends in underweight and obesity from 1990 to 2022: a pooled analysis of 3663 population-representative studies with 222 million children, adolescents, and adults. Lancet.

[2] Limpitikul W.B., Garcia-Contreras M., Spangler P. (2026). Increased arrhythmic risk in obesity is transduced by adipose tissue-derived extracellular vesicles. JACC Basic Transl Sci.

[3] Hinte L.C., Castellano-Castillo D., Ghosh A. (2024). Adipose tissue retains an epigenetic memory of obesity after weight loss. Nature.

[4] Tiash S., Patel S.A., Russo M. (2026). Immune cells regulate circulating adipocyte extracellular vesicle levels in response to metabolic shifts. Cell Metab.

[5] Crewe C., Funcke J.-B., Li S. (2021). Extracellular vesicle-based interorgan transport of mitochondria from energetically stressed adipocytes. Cell Metab.

